# Inelastic mechanical descriptors for osteoporotic hip fracture discrimination with 3D-DXA-based nonlinear finite element models

**DOI:** 10.3389/fbioe.2025.1673339

**Published:** 2025-10-23

**Authors:** Elham Alizadeh, Carlos Ruiz Wills, Luis del Rio, Silvana Di Gregorio, Ludovic Humbert, Jérôme Noailly

**Affiliations:** ^1^ BCN MedTech, Department of Engineering, Pompeu Fabra University, Barcelona, Spain; ^2^ CETIR Ascires, Barcelona, Spain; ^3^ 3D-Shaper Medical, Barcelona, Spain

**Keywords:** biomechanics, osteoporosis, hip fracture discrimination, 3D-DXA, finite element

## Abstract

**Introduction:**

Dual-energy X‐ray absorptiometry (DXA) is the gold standard for diagnosing osteoporosis. Advances in 2D‐3D modelling to generate patient-specific 3D‐DXA models out of DXA images enable accurate volumetric representations of the femur, with potential for fracture risk prediction when combined with finite element (FE) analyses. This study evaluates the ability of 3D‐DXA-based FE models to discriminate hip fractures under side-fall loading.

**Methods:**

We used a retrospective case‐control study including 128 women, 64 of whom suffered a hip fracture. Mechanical descriptors, including strength, nonlinear deformation, residual displacement, and energy absorption under elastic‐plastic assumptions, were derived from force-displacement curves.

**Results:**

The area under the receiver operating characteristic curve (AUROC) showed that strength and trabecular volumetric bone mineral density (vBMD) equally discriminated between fracture and control subjects. Residual displacement due to plastic strain accumulation at failure emerged as a key descriptor which, when combined with strength, significantly improved fracture discrimination (ΔAUROC = 0.11 vs. areal bone mineral density (aBMD); ΔAUROC = 0.08 vs. trabecular vBMD).

**Discussion:**

These findings highlight the potential of 3D-DXA and FE modelling to improve fracture assessment within current DXA-based clinical workflows.

## 1 Introduction

Osteoporosis is identified by a loss of bone mass leading to bone strength reduction. It evolves silently, and its late diagnosis and/or lack of prevention may lead to fragile fractures, loss of life quality, as well as a significant excess of mortality for the patients (Kanis et al., 2021).

Osteoporosis is currently diagnosed by using the areal bone mineral density (aBMD) computed from 2D dual-energy X-ray absorptiometry (DXA) images of the proximal femur and lumbar spine (Stone et al., 2003). However, aBMD is an insufficient metric to estimate the risk of a fragile fracture since it captures neither the distribution of volumetric bone mineral density (vBMD) nor other important factors that control the concentration of mechanical loads in bone tissues, such as bone geometry or external loads. Predictive tools such as FRAX and Garvan (Adler, 2014; Watts et al., 2009; McCloskey et al., 2012) were proposed to estimate the fracture risk, considering different clinical risk factors (age, weight, height, sex, BMI, current smoking status, etc.) combined with aBMD or alone. Although these tools consider multiple risk factors for fracture assessment, they still do not account for bone geometry, spatial distribution of bone density, and mechanical loads. Developing accurate measures of bone strength and mechanical behavior is an essential step toward enhancing fracture risk prediction tools.

In recent studies, subject-specific finite element (FE) models based on quantitative computed tomography (QCT) or DXA scans were proposed, as promising tools to improve fracture risk evaluation, as they allow bone strength estimations by integrating physics-based rationales (Cody et al., 1999). Mechanical experimental models of the proximal femur load and fracture under side-way fall conditions (Dragomir-Daescu et al., 2011; Kok et al., 2021; Courtney et al., 1995; Grassi et al., 2012; Dall’Ara et al., 2016) were developed to validate the aforementioned FE models.

QCT-based subject-specific FE models led to improved fracture risk evaluation compared to aBMD in different studies (Schileo and Taddei, 2021; Fleps and Morgan, 2022). Fleps et al. (2022) used nonlinear QCT-based FE models to assess the fracture risk of the proximal femur under multiple loading conditions by developing an FE modeling pipeline previously established by Enns-Bray et al. (2019). They used nonlinear and linear material models in their study. The results demonstrate that the non-linear FE model was a better approach to discriminate the fracture risk compared to linear FE models. Loading mechanisms with low adduction and internal rotation were found to be more suitable for fracture classification. Cao et al. (2024) presented a QCT-based FE model and computed the yielding load, strength, and energy to failure. They combined the FE parameters with aBMD and six demographical and clinical parameters to develop an FE-based fracture risk index using four loading mechanisms. The results showed that mechanical enrichment of aBMD information through FE parameters improved femoral fracture risk assessment compared to aBMD adjusted for six covariates. Yet, the high cost and radiation dose of QCT limit the clinical applicability of QCT-based FE approaches.

Since DXA is the current clinical standard for osteoporosis management, DXA-based 2D FE models were proposed to predict bone strength (Dall’Ara et al., 2016) and fracture risk in clinical cohorts (Terzini et al., 2019; Leslie et al., 2019; Yang et al., 2018). Dall’Ara et al. (2016) combined experimental investigation on the proximal femur in a standing configuration and a fall onto the greater trochanter, with subject-specific DXA-based 2D linear FE models predicting the bone strength and failure criteria in good agreement with the experimental results. Ulivieri and Rinaudo (2022) introduced the Bone Strain Index (BSI) as a metric for assessing the load resistance of the femur and lumbar spine using DXA-based 2D FE models. Their approach employed linear elastic FE models, where the forces acting on the greater trochanter were determined by the patient’s weight and height. The BSI values represent the average bone strain at the analyzed site (neck BSI and total hip BSI), with higher BSI values indicating increased bone strain and, consequently, a higher fracture risk. Since 2018, several clinical studies have explored the clinical utility of BSI in identifying osteoporotic patient subgroups having a particular tendency to fragility fractures (Rinaudo et al., 2024; Ulivieri et al., 2022; Sornay-Rendu et al., 2022). Naylor et al. (2013) used a DXA-based 2D FE model to assess hip fractures in 728 female subjects. The area under the receiver operating characteristic curve (AUROC) calculated using the load-to-strength ratio (LSR) combined with femoral neck (FN) aBMD was significantly greater compared to FN aBMD alone. Although DXA-based 2D FE simulations could reflect mechanical behaviors (Dall’Ara et al., 2016), their ability for osteoporosis fracture classification remains limited (Naylor et al., 2013). One of the limitations of the DXA-based 2D FE method is the inability to simulate sideways fall models using different loading configurations (Falcinelli et al., 2014). Moreover, in these DXA-based 2D FE models, the volumetric information is missing, and planar information may not sufficiently encompass the out-of-plane distribution of shapes and bone mineral density, depending on the specific characteristics of a patient’s femur.

3D-DXA-based FE models have the potential to overcome the limitations of DXA-based 2D FE models (Thevenot et al., 2014; Väänänen et al., 2015; Grassi et al., 2017; Grassi et al., 2021; Wills et al., 2019). Grassi et al. employed statistical shape and appearance models (SSAM) to reconstruct the three-dimensional anatomy of individual femurs from DXA images, subsequently conducting 3D FE simulations (Grassi et al., 2023). The AUROC values computed using bone strength values normalized to the subject’s weight in 10 different side fall configurations revealed significant improvement compared to aBMD + body mass index (BMI) criteria for hip fracture discrimination (Grassi et al., 2023; Grassi et al., 2025). Although most QCT- and 3D-DXA-based FE models improved fracture risk discrimination compared to aBMD, their implementation in clinical practices remains challenging due to high FE simulation cost, lack of automation (Viceconti et al., 2018), or absence of regulatory approval for use in clinical practice (Grassi et al., 2023). Moreover, assessing a robust mechanical criterion that incorporates the effective mechanical parameters associated with femur fracture could enhance the discrimination of hip fractures in a case-control study.

3D-Shaper^®^ is a software solution that implements the 3D-DXA technology and has obtained regulatory approvals for clinical use (Humbert et al., 2016). Hip DXA scans are analyzed automatically to provide a 3D analysis of the cortical and trabecular compartments. A 3D-DXA-based FE pipeline by using the 3D-Shaper software was used in our previous study (Wills et al., 2019) in which a static peak load that depended on patient mass and height was applied to the femur head. Discrimination of fracture subjects through the local mechanical descriptors was performed in the study, which showed that the major principal stress was a better discriminator compared to the vBMD measured at the same region of interest. Moreover, high correlations were reported between femur strength estimated using the 3D-DXA-based FE model and QCT-based FE models (Dudle et al., 2023; Qasim et al., 2024). The mean computation time per subject for the 3D-DXA algorithm was 1 min 30 s (Humbert et al., 2016) and 15 min for the FE simulation (Qasim et al., 2024), making this approach potentially suitable for use in clinical settings, compared to the other 3D-DXA-based FE algorithms (Grassi et al., 2023).

In the current study, this 3D-DXA-based nonlinear FE model (Qasim et al., 2024) is used to assess the ability of global mechanical descriptors to discriminate between hip fractures and controls. These descriptors integrate the effects of organ geometry, distribution of bone mineral density and mechanical properties, and tissue-dependent organ structure, in 3D patient-specific representations of the proximal femur.

## 2 Materials and methods

### 2.1 Cohort and DXA images

We used data from a retrospective clinical cohort collected in a previous study (Humbert et al., 2020). The retrospective clinical cohort study was performed at CETIR Group Mèdic (Barcelona, Spain) and included 64 subjects with incident hip fractures and 64 controls. The study was conducted under the latest version of the Declaration of Helsinki. Ethical approval was granted by the CETIR Group Mèdic Scientific Committee. Informed consent was obtained from all participants. Subject anonymity was maintained by assigning subject-specific numeric codes to all records. The cohort comprised Caucasian post-menopausal women with baseline visits between 2000 and 2011. Subjects with any prior osteoporotic fractures or diseases other than osteoporosis that affect bone metabolism were excluded from the study. Hip fracture was diagnosed through clinical history or a phone call in case of missing clinical information. A review of clinical history and DXA scans of subjects confirmed the absence of fracture at the baseline and follow-up period in sex- and age-matched subjects in the control group. There was no significant difference ( P -value ≥0.05) between age, height, weight, and body mass index (BMI) between subjects in the hip fracture and control groups (Table 1).

**TABLE 1:** Mean ± standard deviation characteristics in control and hip fracture groups (Humbert et al., 2020).

Characteristics	Control (n = 64)	Hip fractures (n = 64)	${P\;}^{*}$
Age (Years)	68.9 ± 9.0	68.8 ± 8.9	0.975
Height ( cm )	153.2 ± 6.4	153.4 ± 7.3	0.892
Weight ( kg )	62.6 ± 7.9	63.2 ± 11.2	0.722
BMI $\left(\frac{kg}{m^{2}}\right)$	26.7 ± 3.3	26.9 ± 4.6	0.781

^*^P−values from an unpaired two−sample t−test.

DXA scans were conducted using a Prodigy scanner (GE Healthcare, Madison, WI, USA) following the recommendations provided by the manufacturer. aBMD ( $g/cm^{2}$ ), bone mineral content ( g ), and the area ( $cm^{2}$ ), were measured for the entire scanned region of the proximal femur by a trained radiologist using enCORE software v14.10 (GE Healthcare) (Humbert et al., 2020).

### 2.2 3D-DXA analysis

3D-Shaper software v2.14 (3D-Shaper Medical, Barcelona, Spain) was used to analyze the DXA scan and provide patient-specific 3D-DXA geometrical and bone density models. In brief, the software uses an SSAM of the proximal femur, built out of a database of QCT images and registered onto the DXA scan, to estimate a patient-specific 3D femur shape and volumetric density distribution (appearance) (Humbert et al., 2016). This approach allows for representation of both the cortical and trabecular compartments (Humbert et al., 2020), which were subsequently used to generate FE structural meshes with vBMD-based material properties specific to each tissue (Qasim et al., 2024).

Integral vBMD ( $mg/{cm}^{3}$ ) was calculated as the mean vBMD of the cortical and trabecular compartments in the total hip (TH). Trabecular vBMD $\left(mg/{cm}^{3}\right)$ , was calculated as the average vBMD of the trabecular compartment at the TH (Humbert et al., 2016). Cortical surface BMD (sBMD) ( $mg/{cm}^{2}$ ), was computed as the multiplication of the cortical vBMD by the cortical thickness, both averaged over the TH region of interest. The accuracy of the 3D-Shaper modeling methods was evaluated in different studies (Humbert et al., 2016; Dudle et al., 2023). It is noteworthy that in 3D-DXA analyses, the proximal femur, excluding the pelvis and surrounding structures, was considered as the primary region of interest, as it more directly corresponds to the anatomical site relevant for hip fracture risk assessment.

### 2.3 3D-DXA-based nonlinear FE modeling

A 3D-DXA-based nonlinear FE model previously described (Qasim et al., 2024) was utilized to simulate the mechanical behavior of the femur under sideways fall. In brief, the 3D proximal femur anatomy derived from the 3D-Shaper software (Humbert et al., 2016) served for the automatic personalization of FE models. A generic structural FE mesh was morphed into the subject-specific femur shape using thin-plate splines (TPS) transformations. The TPS transformations ensured that separate FE mesh components were consistently morphed into the cortical and trabecular compartments of each subject-specific model. Linear hexahedral eight-node brick elements were used in the FE model.

Using linear interpolations of the bone density values in the image provided by the 3D-Shaper software, the subject-specific bone densities were mapped onto the FE meshed model. In each tissue compartment, the elastic modulus of each element was determined by using the density-elasticity relationships (Ho et al., 1992; Keller, 1994) presented in Equation 1 where the elastic modulus $\left(E\right)$ was in MPa , the ash density $\left(\rho_{ash}\right)$ in $\frac{g}{{cm}^{3}}$ , and the apparent density $\left(\rho_{app}\right)$ in $\frac{kg}{m^{3}}$ .

$$E_{Trabecular}=0.003715\times {\left(\rho_{app}\right)}^{1.96},E_{Cortical}=10200\times {\left(\rho_{ash}\right)}^{2.01}$$ (1)

The Poisson’s ratio was assumed to be 0.3. The ash density of the bone, in $\frac{g}{{cm}^{3}}$ , was calculated as follows (Equation 2) (Schileo et al., 2008a):

$$\rho_{ash}=0.87\rho_{QCT}+0.079$$ (2)

where $\left(\rho_{QCT}\right)$ refers to the radiological density, which was calculated using the vBMD values ( $\frac{g}{{cm}^{3}}$ ) derived from the 3D-Shaper modeling process. To access the apparent density in Equation 1, we assumed $\frac{\rho_{ash}}{\rho_{app}}$ ratio of 0.6, in the range of values (0.55–0.63) provided by available studies, where both $\rho_{ash}$ and $\rho_{app}$ were in $\frac{g}{{cm}^{3}}$ (Schileo et al., 2008a; Goulet et al., 1994; Keyak et al., 1994).

To simulate the nonlinear behavior of bone, an elastic-perfect plastic model was applied (Johannesdottir et al., 2017; Bayraktar et al., 2004; Bevill et al., 2007; Morgan and Keaveny, 2001):

$$\sigma_{yc\;\left(Trabecular\right)}=38.5\times {\left(\rho_{app}\right)}^{1.48},\sigma_{yt\left(Trabecular\right)\;}=22.6\times {\left(\rho_{app}\right)}^{1.26}\;\sigma_{yc\left(Cortical\right)\;}=-041+0.0062\times E_{cortical},\sigma_{yt\left(Cortical\right)\;}=0.33+.0039\times E_{cortical}$$

where $\sigma_{yc\;}$ and $\sigma_{yt\;}$ represent the compressive and tensile yield stress, respectively.

**FIGURE 1:**
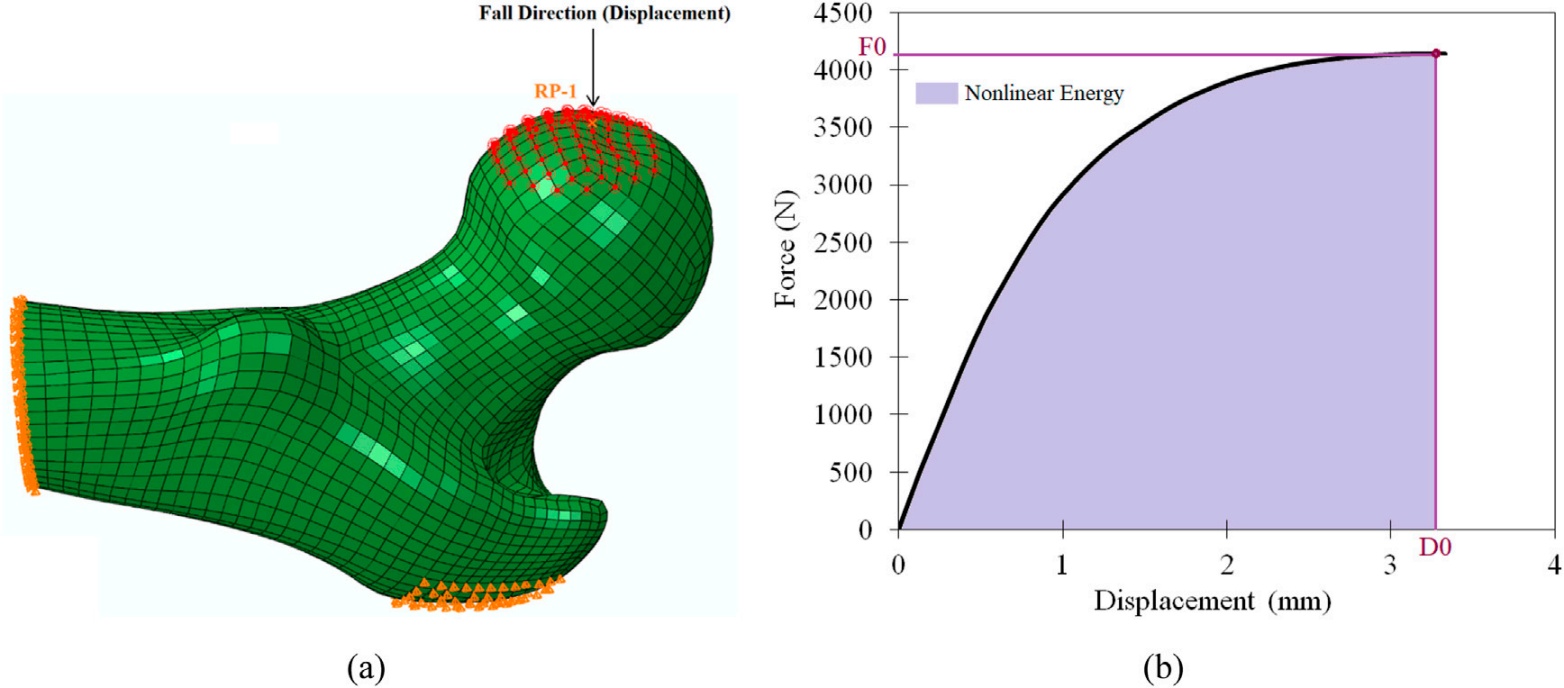
3D-DXA FE femur model under side-way fall direction **(a)** and FE Strength and displacement on force-displacement plot **(b)**.

Boundary conditions that mimic a state-of-the-art experimental model of lateral fall with femur cadaveric experiments (Dragomir-Daescu et al., 2011; Kok et al., 2021; Courtney et al., 1995; Grassi et al., 2012; Dall’Ara et al., 2016), recurrently used in FE models for femur strength calculation (Grassi et al., 2012; Grassi et al., 2023; Abe et al., 2022) were employed in the 3D-DXA-based FE simulations. The end of the modelled shaft region was fully constrained, and the surface of the greater trochanter was fixed only in the fall direction (Figure 1a). A rigid-body constraint imposed a displacement on the femur head through a reference point (RP), until the reaction force started to drop with increasing displacements. Such a drop was considered as a surrogate of mechanical failure, following the nonlinear apparent elastic-plastic response of the femur model. The RP and the corresponding slave nodes on the femur head are shown in Figure 1a. The strength ( $F_{0}$ ) was defined as the corresponding maximum force, and both $F_{0}$ and the corresponding displacement, $D_{0}$ , were used as the main output parameters of the 3D-DXA-based nonlinear FE model (Figure 1b). Furthermore, to account for variability in impact direction, different sideways fall configurations covering 0°–30° of adduction and internal rotation were also modeled in the study (Falcinelli et al., 2014; Grassi et al., 2023).

**FIGURE 2:**
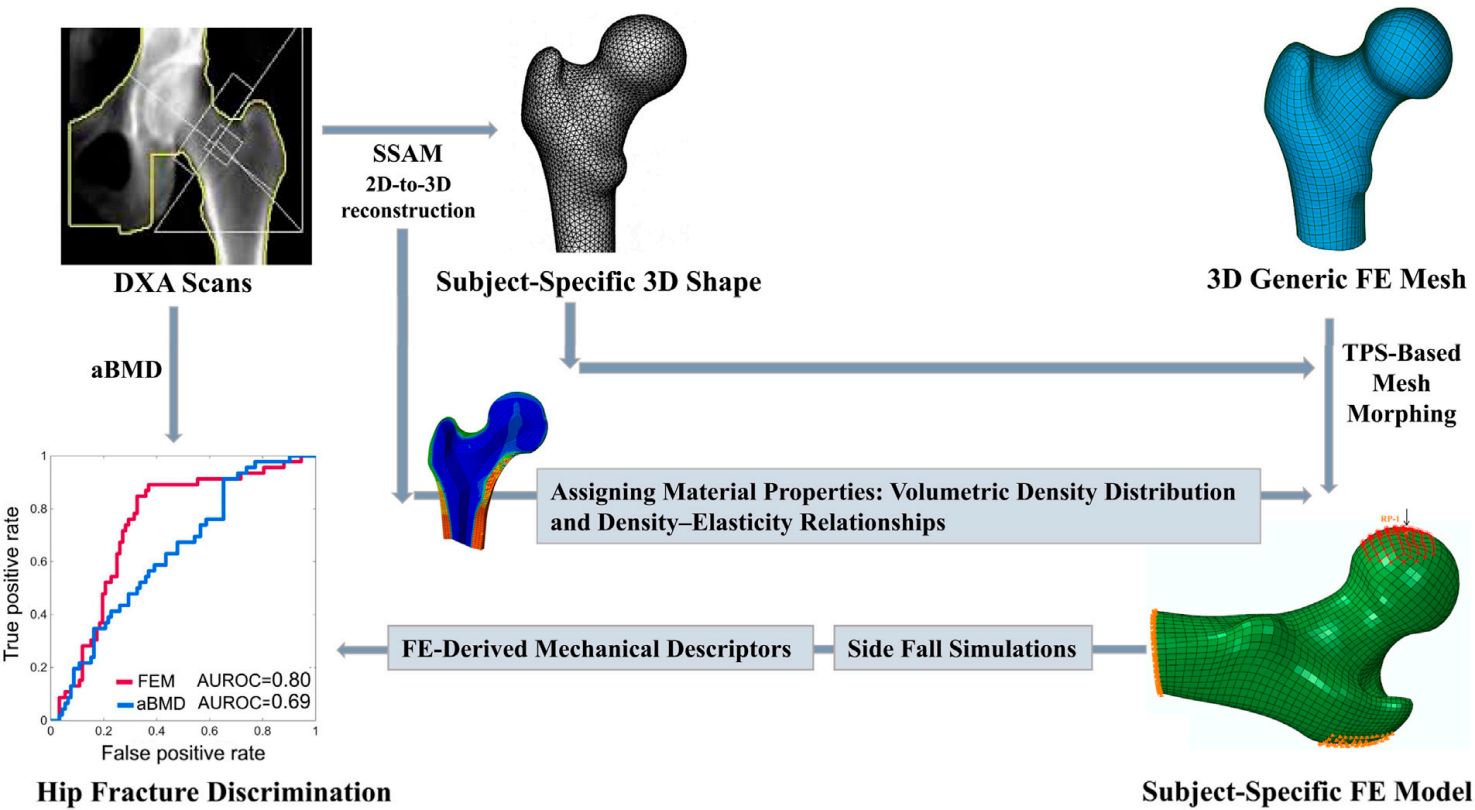
Schematic representation of the concept underlying this study.

Figure 2 provides a schematic representation of the conceptual framework adopted in this study.

### 2.4 Mechanical descriptors for fracture and non-fracture discrimination

The strength ( $F_{0}$ ), displacement at failure ( $D_{0}$ ), and the nonlinear energy up to the failure point (Figure 1b) were used as nonlinear mechanical descriptors for fracture group discrimination.

**FIGURE 3:**
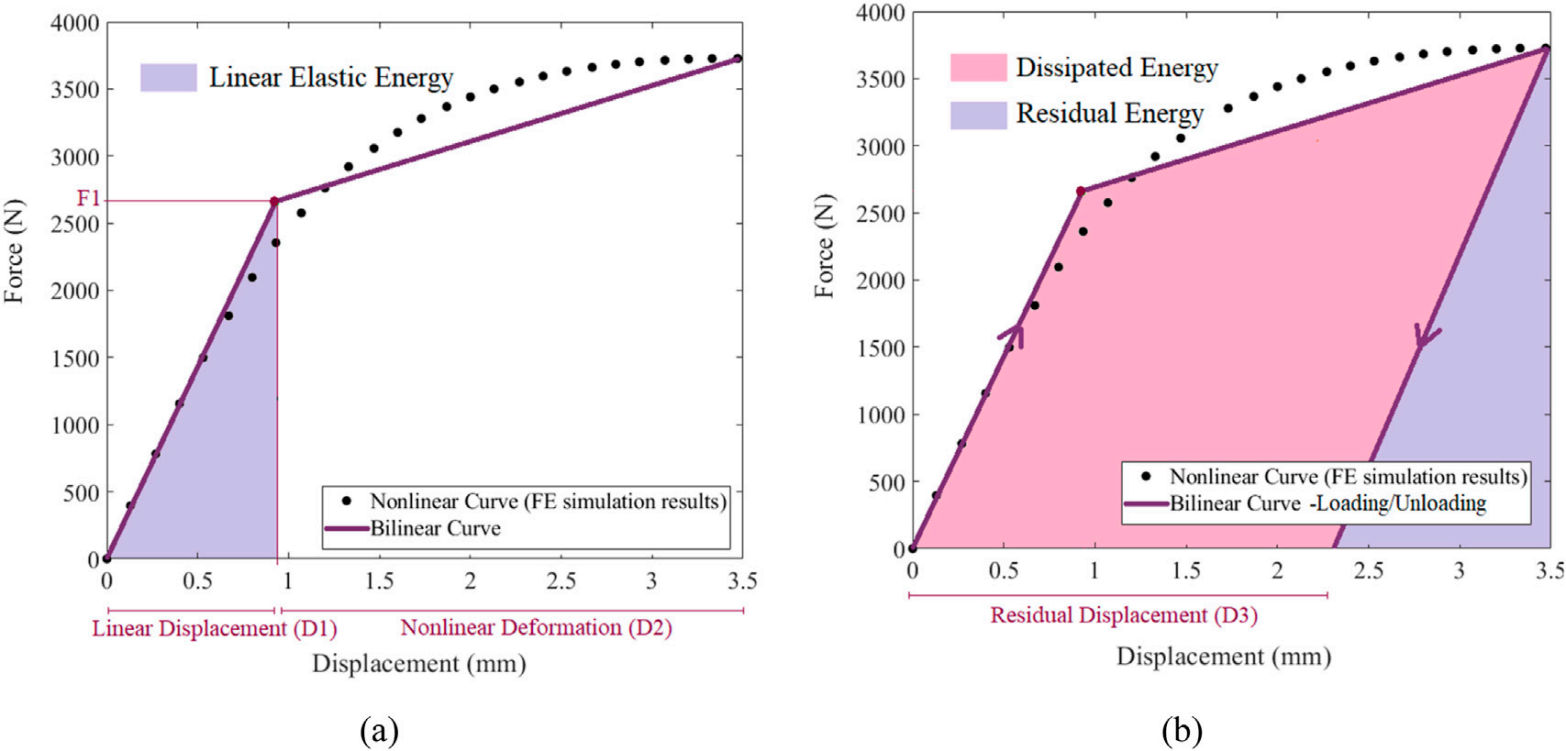
Piecewise linear analysis, **(a)** bilinear force-displacement curve of the femur, **(b)** energy evolution characteristic of the FE analysis.

To quantify the different characteristics of the elastic-plastic behavior, the nonlinear FE-derived curves were post-processed using an optimized piecewise linear method, described in detail in the Supplementary Appendix A. The piece-wise approximation provided a series of additional mechanical descriptors (Figure 3). These included: the linear elastic force ( $F_{1}$ ); the linear elastic displacement ( $D_{1}$ ); the area under the linear elastic region of the bilinear curve (linear elastic energy); the nonlinear deformation ( $D_{2}$ ) (Figure 3a). A loading/unloading analysis was also conducted on the nonlinear FE models to assess the mechanical energy dissipated by the accumulated plastic deformations. Key parameters from this analysis, including residual displacement ( $D_{3}$ ), dissipated energy *per se*, and residual energy (elastic energy), are illustrated in Figure 3b. The full list of mechanical descriptors and combinations thereof, used for the discrimination of fragile fracture in the cohort, is provided in Table 2.

**TABLE 2:** FE-based mechanical descriptors obtained by the nonlinear analysis, piecewise linear analysis, and their combination.

Descriptors			
FE-Based Mechanical Descriptors	Nonlinear FE Analysis		Strength ( $F_{0}$ ), Displacement ( $D_{0}$ ), Nonlinear Energy
	Piecewise Linear Analysis	*Linear Elastic- Based*	Linear Elastic Force ( $F_{1}$ ), Linear Elastic Displacement ( $D_{1}$ ), Linear elastic energy
		*Nonlinear Plastic- Based*	Nonlinear Deformation ( $D_{2}$ ), Residual Displacement ( $D_{3}$ ), Dissipated Energy, Residual Energy (Elastic Energy)
	Combination ofMechanical Descriptors	*Strength-Based- Descriptors*	Strength + Displacement, Strength + Nonlinear Energy, Strength + Nonlinear Deformation, Strength + Residual Displacement, Strength + Dissipated Energy, Strength + Residual Energy
		*Linear Elastic Force- Based Descriptors*	Linear Elastic Force + Linear Elastic Displacement, Linear Elastic Force + Linear elastic energy, Linear Elastic Force + Nonlinear Deformation, Linear Elastic Force + Residual Displacement, Linear Elastic Force + Dissipated Energy, Linear Elastic Force + Residual Energy

Among the analysed parameters, strength is the maximum load sustained by the femur before global failure, reflecting overall bone structural integrity. Displacement at maximum reaction force denotes the volume fraction of elements that have transitioned into the plastic region, indicating the bone’s capacity to deform prior to fracture. Nonlinear energy quantifies the total mechanical energy absorbed up to failure, integrating elastic and plastic contributions, and reflects intrinsic bone toughness. Greater energy absorption is protective, as bone capable of dissipating higher mechanical energy during a fall is less likely to fracture under equivalent strength.

Within the elastic regime, linear elastic force corresponds to the elastic mechanical energy of elements remaining in the elastic region, whereas linear elastic displacement represents their displacement, indicating initial and functional bone deformability. These two parameters characterize bone stiffness; lower force at a given displacement, or greater displacement at a given force, reflects reduced stiffness, linked to thinner cortices or lower trabecular density. Linear elastic energy quantifies the fraction of total mechanical energy absorbed elastically, highlighting the bone’s capacity to resist loading prior to plastic deformation, and reflects its ability to store reversible energy, closely associated with stiffness and load-bearing capacity.

Beyond the elastic region, nonlinear deformation, calculated as the difference between maximum displacement at the failure point and linear elastic displacement, represents the post-yield ductility of the model, potentially mitigating fracture risk despite low bone strength. Residual displacement serves as a surrogate for the elements, i.e., the volume, that have undergone irreversible failure or plastic deformation due to local stress concentrations. Dissipated energy corresponds to mechanical energy due to plastic deformations. Residual energy, i.e., the energy retained after unloading, provided a quantification of how much mechanical energy could remain at failure, according to the strength model.

The maximum load sustained by the femur, captured as strength in the nonlinear analysis and as linear elastic force in the piecewise linear analysis, was further combined with deformation- and energy-based metrics. It aimed to represent a more comprehensive assessment of bone mechanical behaviour, reflecting a personalised balance of stored and dissipated energy along the way to failure. The mechanical description and clinical relevance of *Strength-Based- Combinations* and *Linear Elastic Force- Based Combination* are presented in Supplementary Appendix B (Supplementary Tables SB1, SB2).

### 2.5 Statistical analysis

Logistic regression and AUROC were used in this study to evaluate how the descriptors of the 3D-DXA-based FE model discriminate between fracture subjects and controls, compared to aBMD and 3D-DXA cortical and trabecular parameters. Furthermore, for variables that followed a normal distribution, we applied the unpaired two-sample t-test and for those that did not meet the assumption of normality, we used the non-parametric Mann–Whitney U test to assess whether the mean values of the mechanical parameters in the case group differed significantly from those in the control group. The mechanical descriptors were combined in a multi-logistic regression analysis for fracture assessment. A leave-one-out cross-validation (Airola et al., 2011; Stone, 1974) was used in all logistic regressions, and the statistical significance of the differences among the obtained AUROC values was evaluated using the DeLong method (DeLong et al., 1988).

## 3 Results

### 3.1 Mechanical behavior of the femur under side-way fall configuration

**FIGURE 4:**
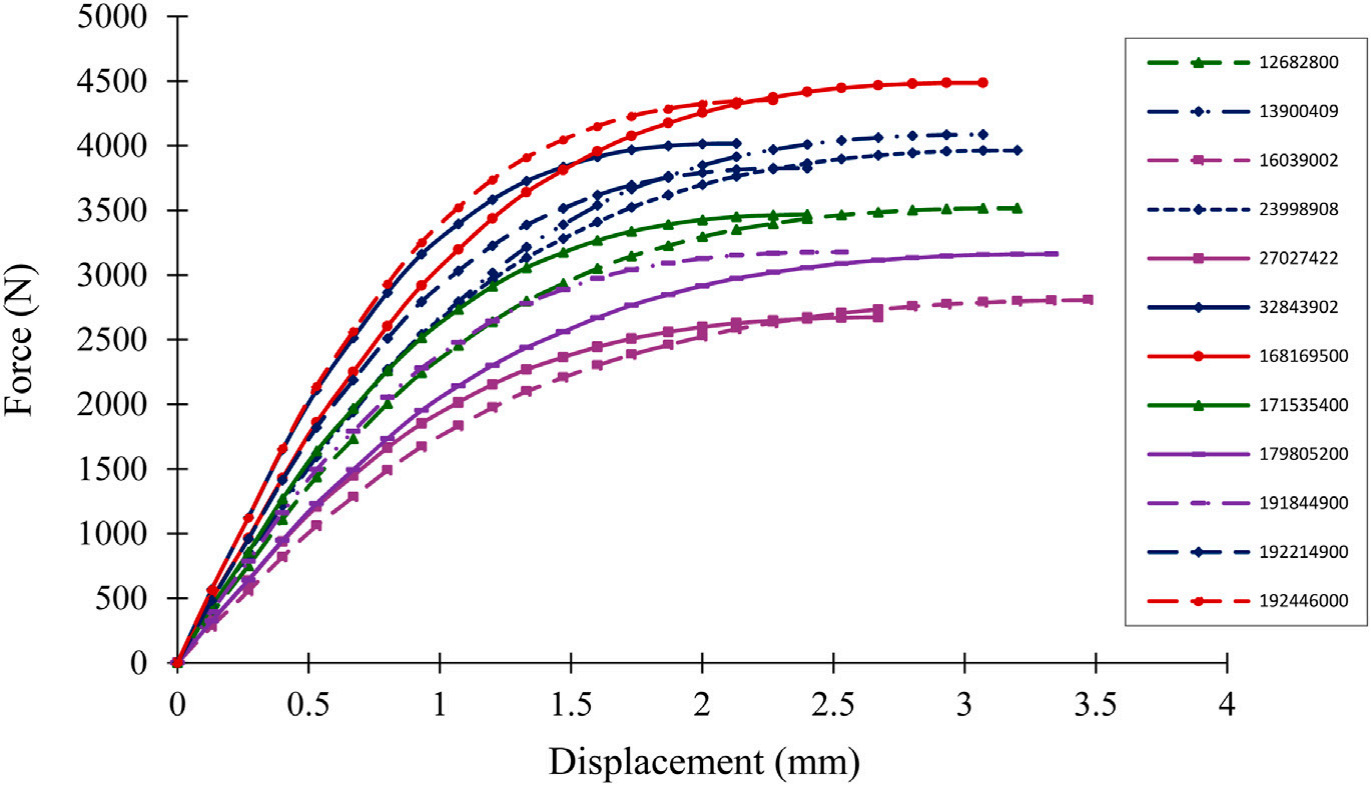
Force-displacement curves of a few subjects under sideway fall FE simulation.

Figure 4 illustrates the force-displacement curves for 3D-DXA-based femur models simulated under side-fall loading, in randomly selected subjects. The curves show that subjects with comparable bone strength could reach different failure points, characterized by varying displacement (D_0_) and nonlinear energy values.

3D-DXA-based FE analysis results, depicting the distribution of maximum principal plastic strain and maximum principal stress at maximum reaction force, in a randomly selected femur model under side-fall loading is provided in the Supplementary Appendix C. High values of both maximum principal plastic strain and stress were observed at the femoral neck, suggesting this region as the most probable site of structural failure.

### 3.2 FE-predicted mechanical descriptors and their ability to discriminate fracture and non-fracture subjects

The mean ± standard deviation of TH aBMD, 3D-DXA measurements, and mechanical descriptors calculated using the FE analysis for the hip fracture and control groups are presented in Table 3. Parameters including aBMD, integral vBMD, trabecular vBMD, cortical sBMD, strength, linear elastic force, linear elastic energy, and residual energy, were significantly lower in hip fracture cases compared to controls. In contrast, displacement, linear elastic displacement, nonlinear deformation, and residual displacement were significantly higher in hip fracture cases. Nonlinear energy was higher in controls, while dissipated energy was greater in hip fracture cases, although none of these differences were statistically significant.

**TABLE 3:** Mean ± standard deviation of DXA, 3D-DXA measurement, and FE-based mechanical descriptors in the control and hip fracture groups.

Methods			Parameters	Control	Hip fracture	${P\;}^{*}$
DXA (TH)			aBMD $\left(\frac{g}{{cm}^{2}}\right)$	0.85 ± 0.12	0.76 ± 0.11	<0.001
3D-DXA Measurement (TH)			Integral vBMD $\left(\frac{mg}{{cm}^{3}}\right)$	290.17 ± 47.92	252.23 ± 41.22	<0.001
			Trabecular vBMD $\left(\frac{mg}{{cm}^{3}}\right)$	140.39 ± 34.94	112.2 ± 28.54	<0.001
			Cortical sBMD $\left(\frac{mg}{{cm}^{3}}\right)$	144.13 ± 18.90	131.30 ± 18.21	<0.001
FE-Based Mechanical Descriptors	Nonlinear FE Analysis		Strength (N)	3407.70 ± 568.3	2984.82 ± 552.81	<0.001
			Displacement (mm)	2.67 ± 0.28	2.98 ± 0.31	<0.001
			Nonlinear Energy (N.mm)	6588.48 ± 1197.23	6504.74 ± 1248.04	0.69
	Piecewise Linear Analysis	*Linear Elastic- Based*	Linear Elastic Force (N)	2707.66 ± 469.18	2351.17 ± 446.46	<0.001
			Linear Elastic Displacement (mm)	0.92 ± 0.06	0.97 ± 0.06	<0.001
			Linear elastic energy (N.mm)	1250.07 ± 216.58	1139.39 ± 211.78	**0.004**
		*Nonlinear Plastic- Based*	Nonlinear Deformation (mm)	1.75 ± 0.23	2.01 ± 0.28	<0.001
			Residual Displacement (mm)	1.50 ± 0.21	1.74 ± 0.23	<0.001
			Dissipated Energy (N.mm)	4579.60 ± 875.13	4621.86 ± 885.90	0.78
			Residual Energy (N.mm)	1983.11 ± 335.87	1839.58 ± 342.28	**0.01**

^*^ P −values from unpaired two-sample t-test for normally distributed parameters or Mann–Whitney U test for non-normally distributed parameters, the bold values denote statistically significant results.

The AUROCs achieved with the FE–based mechanical descriptors were compared to those achieved with aBMD and 3D-DXA measurements, i.e., integral vBMD, trabecular vBMD, and cortical sBMD (Table 4). The FE-based mechanical descriptors were classified into four categories: (i) Nonlinear FE Analysis; (ii) Piecewise Linear Analysis; (iii) Combination of Bone Strength with Mechanical Descriptors; (iv) Combination of Linear Elastic Force with Mechanical Descriptors.

1. *Nonlinear FE Analysis*: Nonlinear FE analysis identified displacement as a key mechanical descriptor for fracture discrimination. However, strength alone showed slightly higher discrimination power compared to aBMD and comparable performance to trabecular vBMD.
2. *Piecewise Linear Analysis:* Piecewise linear analysis generated two categories of mechanical descriptors based on elastic-plastic characteristics: a) linear elastic-based descriptors, including elastic force, displacement, and energy; b) nonlinear plastic-based descriptors, such as nonlinear deformation, residual displacement, and residual energy. Mechanical descriptors derived from these analyses, particularly nonlinear deformation and residual displacement, demonstrated a statistically significant enhancement in discriminating hip fracture cases from controls compared to the aBMD method [AUROC: 0.79 versus 0.69]. Additionally, linear elastic force and linear elastic displacement provided a discrimination capacity similar to trabecular vBMD [AUROC:0.72].
3. *Combination of Bone Strength with Mechanical Descriptors:* Combining FE strength with corresponding displacement, nonlinear energy, and nonlinear plastic-based descriptors significantly enhanced fracture discrimination compared to BMD-based methods. Notable combinations that significantly over-performed any aBMD-based descriptors included: Strength + Displacement, Strength + Nonlinear Energy, Strength + Nonlinear Deformation, Strength + Residual Displacement, and Strength + Dissipated Energy. Among these, FE strength + Residual Displacement emerged as the most effective combination, achieving a notable improvement in AUROC compared to both aBMD (0.80 vs. 0.69, P = 0.001) and trabecular vBMD (0.80 vs. 0.72, P = 0.016).
4. *Combination of Linear Elastic Force with Mechanical Descriptors:* Integrating linear elastic force with the corresponding linear elastic displacement, energy, and nonlinear plastic-based descriptors further improved the ability to discriminate fracture cases from controls across most combinations. Particularly effective combinations included: Linear Elastic Force + Linear Elastic Displacement, Linear Elastic Force + Linear Elastic Energy, Linear Elastic Force + Nonlinear Deformation, Linear Elastic Force + Residual Displacement, and Linear Elastic Force + Dissipated Energy. Notably, Linear Elastic Force + Residual Displacement showed a statistically significant improvement in fracture discrimination compared to aBMD and 3D-DXA measurements [0.79 versus 0.69 and 0,72, respectively], further highlighting the importance of incorporating nonlinear-plastic based descriptors into fracture risk models.

**TABLE 4:** AUROC calculated using leave-one-out cross-validation.

Methods			Descriptors	AUROC	P -value^*^	
					(Versus aBMD)	(Versus trabecular vBMD)
DXA			aBMD	**0.69**	-	0.192
3D-DXA measurement			Integral vBMD	0.71	0.206	0.433
			Trabecular vBMD	**0.72**	0.192	-
			Cortical sBMD	0.66	0.129	0.088
FE-Based Mechanical Descriptors	Nonlinear FE Analysis		Strength	0.72	0.275	0.994
			Displacement	0.78	0.057	0.14
			Nonlinear Energy	0.52	0.0006	0.0004
	Piecewise Linear Analysis	*Linear Elastic- Based*	Linear Elastic Force	0.72	0.230	0.959
			Linear Elastic Displacement	0.72	0.583	0.979
			Linear elastic energy	0.65	0.255	0.116
		*Nonlinear Plastic- Based*	Nonlinear Deformation	**0.79**	**0.039**	0.099
			Residual Displacement	**0.79**	**0.033**	0.085
			Dissipated Energy	0.51	**0.035**	**0.0083**
			Residual Energy	0.63	0.162	0.078
	Combination of Mechanical Descriptors	*Strength-Based- Descriptors*	Strength + Displacement	**0.79**	**0.002**	**0.022**
			Strength + Nonlinear Energy	**0.79**	**0.003**	**0.030**
			Strength + Nonlinear Deformation	**0.79**	**0.002**	**0.026**
			Strength + Residual Displacement	**0.80**	**0.001**	**0.016**
			Strength + Dissipated Energy	**0.79**	**0.002**	**0.022**
			Strength + Residual Energy	0.73	0.100	0.621
		*Linear Elastic Force- Based Descriptors*	Linear Elastic Force + Linear Elastic Displacement	0.74	**0.046**	0.378
			Linear Elastic Force + Linear elastic energy	0.74	0.061	0.468
			Linear Elastic Force + Nonlinear Deformation	0.78	**0.010**	0.074
			Linear Elastic Force + Residual Displacement	**0.79**	**0.005**	**0.033**
			Linear Elastic Force + Dissipated Energy	0.77	**0.014**	0.103
			Linear Elastic Force + Residual Energy	0.71	0.467	0.841

^*^P-Values calculated between AUROCs using the Delong method; the bold values denote statistically significant results; Bold AUROC values indicate the highest values within each group of descriptors; aBMD, integral vBMD, and cortical sBMD, were calculated at the TH, region of interest.

**FIGURE 5:**
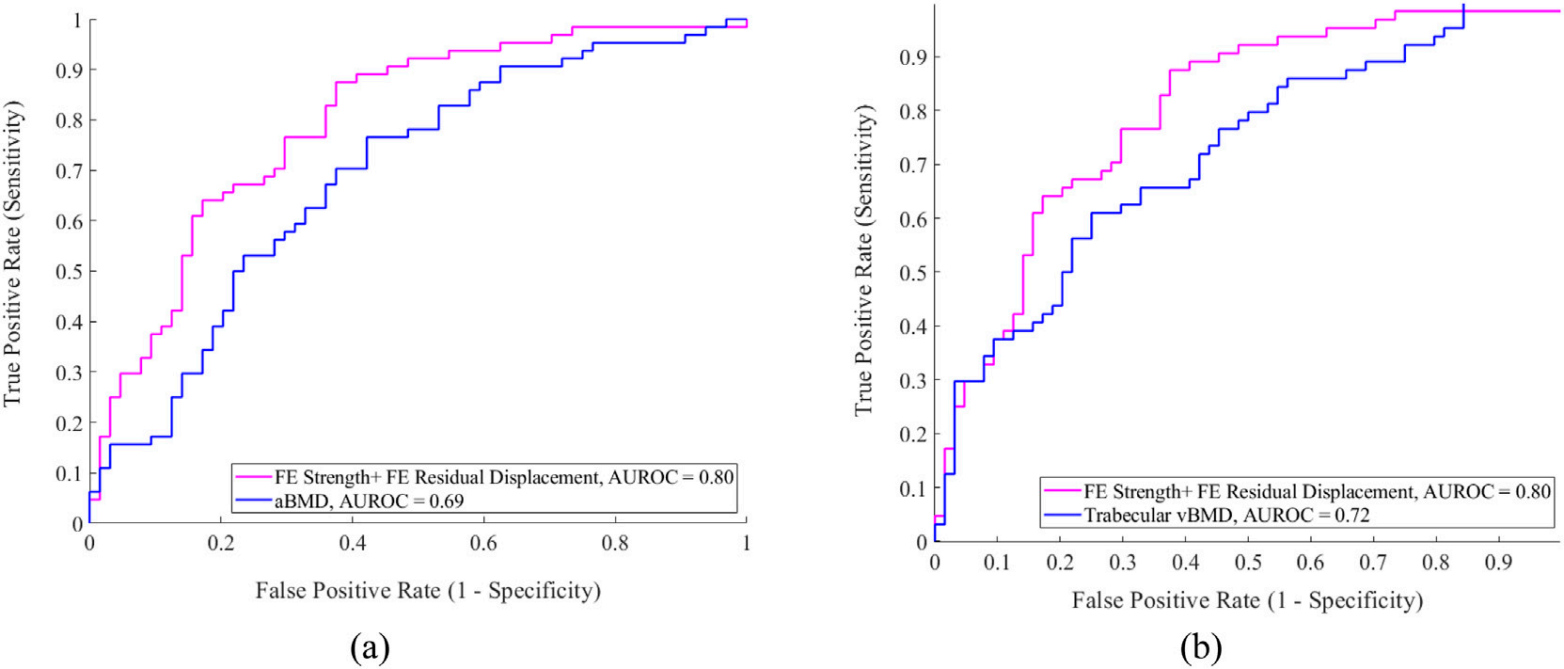
ROC curves for the mechanical descriptors compared with **(a)** aBMD and **(b)** Trabecular vBMD.

The ROC curve obtained using strength + residual displacement, which resulted in the highest AUROC, is presented in Figure 5, together with the ROC curves obtained using aBMD and trabecular vBMD. The FE-derived mechanical descriptors for the control and fracture subjects are presented in Supplementary Appendix D (Supplementary Tables SD1, SD2). It is noteworthy that the influence of different fall scenarios and the impact thereof on the discrimination ability is summarized in Supplementary Appendix E. The presented scenarios correspond to those most commonly applied in previous investigations (Falcinelli et al., 2014; Grassi et al., 2023; Pinilla et al., 1996; Abe et al., 2022). Remarkably, the capacity of the femur strength parameter derived from FE simulations to overcome the capacity of the discrimination by aBMD (AUROC = 0.69) depended on the loading direction. Strength AUROC values ranged from 0.68 to 0.72. The best performance of the strength descriptor was achieved with a load aligned with the vertical when the fall models had no internal rotation and no adduction. In every case, Strength AUROC values were overperformed when mechanical information about the inelastic model response was considered for discrimination, according to the best-performing descriptors achieved with the default load.

## 4 Discussion

This study explored the added value of the global mechanical descriptors to discriminate the occurrence of femur fragile fracture in a case-control study using DXA-based 3D modeling and FE methods.

Three sets of FE-derived descriptors were chosen to capture distinct mechanical and possibly clinically meaningful aspects of hip fracture risk, as far as fracture risk groups can be compared. Importantly, in contrast to sophisticated FE models that describe bone failure mechanics (Zysset et al., 2015), we propose mechanical surrogates as per the capacity of the femoral bone to absorb mechanical energy in a binary partition of osteoporosis patients: fracture cases, and controls. In our models, nonlinear FE-based descriptors, extracted from the load-displacement curve up to failure, capture the volumetric accumulation of plastic strain up to the point at which the femur model stops to increasingly resist the load. We see from our results (Table 4, Strength-based + nonlinear descriptors) that the nonlinearity associated with this simulated process nicely complements the state-of-the-art bone strength, to eventually overperform the discrimination capacity of both DXA-based aBMD and 3D-DXA-based vBMD. The piecewise modelling of the nonlinear mechanical response further informed about how a specific femur model was reaching its maximum ability to store mechanical energy: it was used to evaluate the balance of elastic energy stored vs. dissipating plastic deformations until the maximum reaction force was reached. Especially, it gave access to the residual displacement descriptor that, once combined with the strength, led to the best discrimination capacity, and proved to be, *per se* a promising descriptor for fracture risk assessment. Overall, these results, achieved with surrogates of the inelastic behaviour of bone, suggest that quantifying the bone’s post-yield behaviour, according to measurable bone properties at baseline, could improve significantly the clinical assessment of the risk of bone fragile fracture.

The proposed analyses underscore two critical aspects of fracture risk assessment. First, relying on bone strength alone is insufficient for patient-specific fracture discrimination, as it overlooks critical mechanical parameters combined with the elastic-plastic assumptions, hereby subjacent to the concept of strength. Second, the FE analyses in this study use a static loading scenario, whereas fatigue effects from repetitive or dynamic loads shall influence fracture risk through micro-damage accumulation (Mirzaali et al., 2020), especially when the bone turnover balance is altered, as in osteoporosis. Fatigue-related effects cannot be addressed by using bone strength alone, but we hypothesized that their representation can be improved through parameters such as residual displacement, nonlinear deformation, and energy absorption. Such assessment of the FE-predicted mechanical parameters, in the present study, considers cross-validation, which is otherwise too often overlooked in the literature.

To assess the discrimination power and clinical applicability of the proposed mechanical descriptors, we compare those with existing FE-based methods, including QCT-based, 2D-DXA-based, and 3D-DXA-based models. The discrimination capability of QCT-based FE analysis for hip fractures was first investigated in the MrOS study by Orwoll et al. (2009), which revealed that femoral strength (AUROC:0.83) and the load-to-strength ratio (AUROC:0.79) were key predictors, showing comparable performance with aBMD (AUROC:0.85), without any significant difference. Subsequent studies by Amin et al. (2011) in community-dwelling adults and Kopperdahl et al. (2014) used the AGES Reykjavik cohort and confirmed these findings, showing no significant improvement using FE-based parameters compared to aBMD. In contrast, our study demonstrates that the proposed FE strength + residual displacement descriptor from the 3D-DXA-based model provides superior fracture discrimination compared to aBMD and outperforms previous QCT-based FE studies (Orwoll et al., 2009; Amin et al., 2011; Kopperdahl et al., 2014). A large retrospective study by Adams et al. (2018), following the MrOS (Orwoll et al., 2009) and AGES Reykjavik (Kopperdahl et al., 2014) protocols, provided the first evidence of a significant advantage for QCT-based FE analysis (hazard ratio (HR) = 2.8, 95% CI 2.2–3.5) over aBMD (HR = 2.1, 95% CI 1.7–2.5), validating the clinical value of the Biomechanical Computed Tomography (BCT) approach. Keaveny et al. (2023) introduced BCT^+^, an advanced method incorporating nine variables, including bone strength, age, bone volume, fat content, and body composition. BCT^+^ outperformed BMD and FRAX, achieving an AUROC of 0.79 vs. 0.72 and 0.73, respectively. Our study showed a greater improvement in incident hip fracture assessment (ΔAUROC = 0.11 vs. 0.07), and importantly, our 3D-DXA-based FE models are verified against QCT-based FE models (Dudle et al., 2023; Qasim et al., 2024). Bessho et al. (2007) introduced a novel concept, later expanded by Falcinelli et al. (2014), who assessed femoral strength under various loading conditions to identify the weakest scenario for each patient. Their findings showed that minimum femoral strength better-discriminated fractures than standard sideways fall strength, achieving a higher AUROC than aBMD (0.88 vs. 0.79). However, the study had limitations, as QCT images were acquired post-fracture by scanning the contralateral femur, potentially affecting FE model accuracy in fracture risk assessment. Fleps et al. (2022) demonstrated that nonlinear FE models improved fracture prediction over aBMD (AUROC: 0.78 versus 0.72 in men, 0.75 versus 0.69 in women) within the AGES Reykjavik cohort. In their study, the most discriminative fall direction had low internal rotation and adduction, differing from Falcinelli et al. (2014). The AUROC values in our study were slightly higher than those reported by Fleps et al. (2022), further emphasizing the clinical potential of the 3D-DXA-based FE model alternative. Although QCT-based FE models show strong fracture discrimination (Adams et al., 2018; Keaveny et al., 2023), their high cost and radiation exposure limit widespread use. In addition, while most previous QCT-based FE studies focused on single metrics such as femoral strength, the load-to-strength ratio, or the multivariate BCT^+^ combining strength with clinical parameters, none have explored novel mechanical descriptors from FE simulations. Our work introduces descriptors integrating strength with nonlinear behaviour (e.g., residual displacement), capturing aspects of bone fragility beyond traditional measures. By rigorously assessing their discrimination performance, our findings extend existing QCT-based approaches and highlight the potential of global mechanical descriptors to improve fracture-risk prediction.

As an alternative, DXA-based FE models have been proposed to estimate femoral strength for hip fracture assessment (Wills et al., 2019; Grassi et al., 2023). DXA-based 2D FE models have been developed to assess bone strength and hip fracture risk. Naylor et al. (2013) found only marginal improvement in fracture discrimination using strength (AUROC = 0.67) and load-to-strength ratio (AUROC = 0.68) compared to FN aBMD (AUROC = 0.66), with a slight increase after adjustment for FN aBMD (AUROC = 0.68 and 0.69, for FN aBMD + strength and FN aBMD + load-to-strength ratio, respectively). Yang et al. (2014) reported femoral strength as a strong predictor of hip fracture risk after adjusting for age and BMI (HR = 2.2, 95% CI 1.95–2.50), outperforming TH aBMD (HR 1.9, 95% CI 1.7–2.1) but not FN aBMD (HR 2, 95% CI 1.8–2.3). More recently, DXA-derived indexes, such as the Bone Strain Index (BSI), have been developed. Sornay-Rendu et al. (2022) utilized a DXA-based 2D FE model to assess FN and TH BSI, alongside aBMD, in the OFELY study, which focuses on all major osteoporotic fractures instead of purely on incident hip fractures like in the present study. The AUROC for TH BSI (0.64) was not significantly different from that of TH aBMD (0.65) or Trabecular Bone Score (TBS) (0.67). In the current study, the discrimination power of aBMD, with an AUROC of 0.69, aligns with those results, and 3D-DXA vBMD tends to improve the discrimination (AUROC = 0.72). These outcomes reveal the likely importance of 3D information, especially if the latter is extended to mechanical descriptors (Table 4).

Indeed, since 2D models do not capture variations in bone geometry, density distribution, or impact forces, DXA-based 3D modeling has been developed to enable 3D-DXA-based FE models, as utilized in the current study (Wills et al., 2019; Grassi et al., 2023). Grassi et al. (2023) employed a 3D-DXA-based linear FE model in a MrOS cohort, demonstrating a significant improvement in the AUROC values for bone strength normalized by body weight (BW) across 10 different fall scenarios compared to BMD combined with BMI (0.78 vs. 0.72). This study is the only 3D-DXA-based investigation directly comparable to our approach and the first to show a statistically significant improvement in hip fracture discrimination over TH aBMD using subject-specific 2D-to-3D FE models. The current study exhibits slightly improved discrimination power compared to Grassi et al. (2023) (ΔAUROC = 0.11 vs. 0.06), likely due to key methodological differences. In contrast to the linear elastic approach adopted by the Authors (Grassi et al., 2023), we incorporated nonlinear material properties, providing a computationally efficient way to reflect the inelastic femur mechanics under side-fall loading, consistent with experimental findings 7,9. Fleps et al. 14 also demonstrated the superiority of nonlinear FE models in fracture discrimination. Besides, (Grassi et al., 2023) estimated femoral strength using a surface-strain criterion (Schileo et al., 2014; Schileo et al., 2008b) originally established from experimental observations (Grassi et al., 2012). While this approach restricts failure assessment to the bone surface, our methodology captures the volumetric behavior of the femur, not excluding that failure prediction might be controlled by the mechanics of internal compartments of the model (Wills et al., 2019). This provides a more comprehensive representation of bone mechanics, including the mechanics of the entire neck and intertrochanteric regions, facilitates clinical translation, and is consistent with the framework proposed by Keaveny et al. (2023), Orwoll et al. (2009), Amin et al. (2011).

Furthermore, our mechanical descriptors enhance patient-specific assessments by integrating organ geometry, BMD distribution, mechanical properties, and structural characteristics. In particular, residual displacement, nonlinear deformation, and energy absorption seemed to capture key determinants of bone fragility between the two patient groups, beyond bone strength alone, ultimately improving fracture prediction.

Extending the above observations, the effect of different fall scenarios was evaluated in the present study, and our findings are consistent with those of Fleps et al. (2022), who reported that fall models with low internal rotation and adduction are the most suitable for discriminating hip fracture cases from controls. Remarkably, the discrimination achieved by incorporating descriptors of the inelastic behaviors of the femur models was consistently superior to that achieved with only the state-of-the-art Strength parameter. Nevertheless, we found a high variability as per the performance of the mechanical descriptor to discriminate the fracture cases, versus the BMD parameters, when load directions were varied (Supplementary Appendix E, Supplementary Table SE1). Therefore, we recommend that probabilistic fracture risk be assessed across a range of fall directions (Falcinelli et al., 2014; Grassi et al., 2023).

The proposed 3D-DXA-based approach offers a clinically viable solution for fracture risk assessment by utilizing standard DXA scans, eliminating additional radiation exposure, and reducing costs compared to QCT-based FE methods (Fleps et al., 2022; Adams et al., 2018). Its computational efficiency, implemented using the commercially available 3D-Shaper software (Humbert et al., 2016), further enhances its clinical feasibility. Processing time per subject to compute 3D-DXA analysis using 3D-Shaper software is 1 min 30 s on an Intel Core i7-4790K CPU, which is significantly faster than the methods used by Grassi et al. (2023), which required approximately 6 hours per scan using 20 CPU cores. Additionally, our FE simulation takes about 15 min per subject using 4 CPU cores, compared to 2 hours reported by Fleps et al. (2022). The existing regulatory approvals for the 3D-Shaper software are also an asset for the future clinical implementation of the 3D-DXA-based FE approach evaluated in this study.

Despite its advantages, the proposed approach has some limitations. The study was based on a retrospective cohort of 64 hip fracture cases and 64 controls. Larger, prospective cohorts are needed for further validation. The elastic-plastic model used to capture inelastic bone mechanics was a rough approximation of the real bone failure mechanics. It allowed computationally affordable simulations and indicated a robust improvement compared to BMD measure or strength calculations alone. Yet, whether macroscopic or multiscale bone failure mechanics models can further improve fracture risk predictions remains to be explored. A challenge also arises in femoral head density reconstruction, as clinical DXA scans often suffer from acetabulum superimposition. This region is excluded from the 2D-to-3D reconstruction using 3D-Shaper software (Humbert et al., 2016), and the femoral head is reconstructed by mapping QCT-derived density onto the DXA image (Humbert et al., 2016). This approximation may affect the accuracy of the femoral geometry and material properties, but rigid-body constraints with a reference point were applied during the FE simulation to mitigate the effect, prevent stress concentration in the femur head, and lead to a more realistic model. The present study prioritized global over local mechanical descriptors (Wills et al., 2019). While this can be considered as a limitation, as local descriptors might achieve improved discriminations (Wills et al., 2019), we hereby avoid the need for site-specific fracture analyses. This strategy reduces sensitivity to mesh convergence, enhances computational efficiency, and reduces the complexity of the mechanical analysis (only one surrogate descriptor for bone fracture risk at the organ level). It gathers, therefore, important characteristics to make the clinical translation accessible. Indeed, the current cohort does not include annotations of fracture location (neck vs. trochanteric). Hence, the possibility to adopt global descriptors fits well with the reality of the data.

## 5 Conclusion

This case-control study demonstrates a significant improvement in hip fracture discrimination using 3D-DXA-based FE models compared to standard aBMD and 3D-DXA measurements. The consideration of the global balance of elastic and inelastic femur mechanics during side-fall simulations was a cornerstone to achieve improved fragile fracture discrimination at reduced computational cost. These findings suggest that 3D-DXA-based FE models could enhance osteoporosis management while maintaining DXA as the clinical standard of care. Among the mechanical descriptors evaluated, the combination of FE strength and residual displacement emerged as the most effective for discriminating hip fractures, as it incorporates key mechanical parameters relevant to hip fracture risk.

## Data Availability

The data analyzed in this study is subject to the following licenses/restrictions: The data are not publicly available due to privacy or ethical restrictions. Requests to access these datasets should be directed to Ludovic Humbert, ludovic.humbert@3d-shaper.com.
