# Supplementary material for: Inelastic mechanical descriptors for osteoporotic hip fracture discrimination with 3D-DXA-based nonlinear finite element models
Source: Front Bioeng Biotechnol. 2025 Oct 23;13:1673339. doi: 10.3389/fbioe.2025.1673339 (PMC12589097; doi:10.3389/fbioe.2025.1673339)
Supplement: Supplementary file 1 [file DataSheet1.docx]

**Appendix A**

An optimized piecewise linear method was applied to the nonlinear force-displacement data from the FE simulation to discriminate hip fracture cases from controls.

We assumed the force-displacement curve to be bilinear. The intersection point of the two linear pieces was identified through optimization with a Rosenbrock function that minimizes the difference between the FE-calculated force-displacement data points and the fitted linear segments. The Rosenbrock function is presented in Eq. (A1) in which $x_{1}$ and $x_{2}$demonstrate the FE-calculated force-displacement data points and the fitted linear segments, respectively.

| $f\left( x \right)=100 {({x_{2}}^{2}-x_{1})}^{2}+{(1-x_{1})}^{2}$ | (A1) |
| --- | --- |

The optimization was constrained by imposing: 1) the initial stiffness, according to the first converged FE solver increments; 2) the final force-displacement point calculated by the FE solver; 3) the area under the piecewise linear (i.e., mechanical energy) curve should equal the area under the nonlinear force-displacement results returned by the FE solver. Calculations were done through an in-house MATLAB code.

**Appendix B**

Tables B1 and B2 present the explanation of Strength-Based Combinations and Linear Elastic Force-Based Combinations.

Table B1. Strength-Based Combinations

| Descriptors | Biomechanical dimension | Clinical/functional relevance |
| --- | --- | --- |
| Strength + Displacement | Integrates ultimate load with deformation at failure, combining resistance and ductility | Identifies brittle bones that fail due to limited deformation despite normal strength, a mechanical parameter linked to skeletal fragility |
| Strength + Nonlinear Energy | Couples failure load with total energy absorbed (elastic + plastic) | Highlights bones with low toughness that fracture easily, even if the peak load is adequate, consistent with a fragility fracture |
| Strength + Nonlinear Deformation | Links ultimate strength with post-yield ductility | Identifies bones with low capacity to redistribute stresses after yielding, a key descriptor of osteoporotic fragility |
| Strength + Residual Displacement | Combines failure load with irreversible plastic deformation after unloading | Reflects accumulation of plastic strain and reduced recovery capacity, relevant for fragility under repetitive loading (e.g., walking, stair climbing) |
| Strength + Dissipated Energy | Couples strength with energy lost through plastic deformation | Reflects the bone’s capacity to absorb or dissipate impact energy during a fall; an imbalance may increase fracture susceptibility |
| Strength + Residual Energy | Links strength with the fraction of energy retained after unloading | Provides insight into post-yield alterations of stiffness and energy storage, linking structural changes to fracture risk |

Table B2. Linear-Elastic-Force-Based Combinations

| Descriptors | Biomechanical dimension | Clinical/functional relevance |
| --- | --- | --- |
| Linear Elastic Force + Linear Elastic Displacement | Relates elastic load to deformation, characterizing stiffness under physiological load | Helps identify bones with reduced stiffness (e.g., cortical thinning, trabecular loss) before yielding, early indicators of osteoporotic bone fragility |
| Linear Elastic Force + Linear Elastic Energy | Integrates peak elastic load with stored reversible energy | Describes the bone’s capacity to sustain physiologic loading without entering the plastic regime, relevant for daily activity tolerance |
| Linear Elastic Force + Nonlinear Deformation | Combines elastic resistance with post-yield ductility | Detects bones that are stiff but prone to sudden failure once yielding begins, a clinically important marker of brittle fracture |
| Linear Elastic Force + Residual Displacement | Relates elastic strength to irreversible deformation after unloading | Highlights bones with early stiffness loss and cumulative plastic damage, reflecting progressive osteoporotic deterioration |
| Linear Elastic Force + Dissipated Energy | Merges elastic stiffness with plastic energy dissipation | Assesses how efficiently bone transitions from elastic to plastic behavior; inadequate dissipation is linked to increased fracture risk |
| Linear Elastic Force + Residual Energy | Links elastic capacity with energy retained after unloading | Reflects damage accumulation and reduced recovery potential following failure loading |

**Appendix C**

Fig. C1 demonstrates 3D-DXA-based finite element results for a randomly selected femur under lateral fall loading.

| 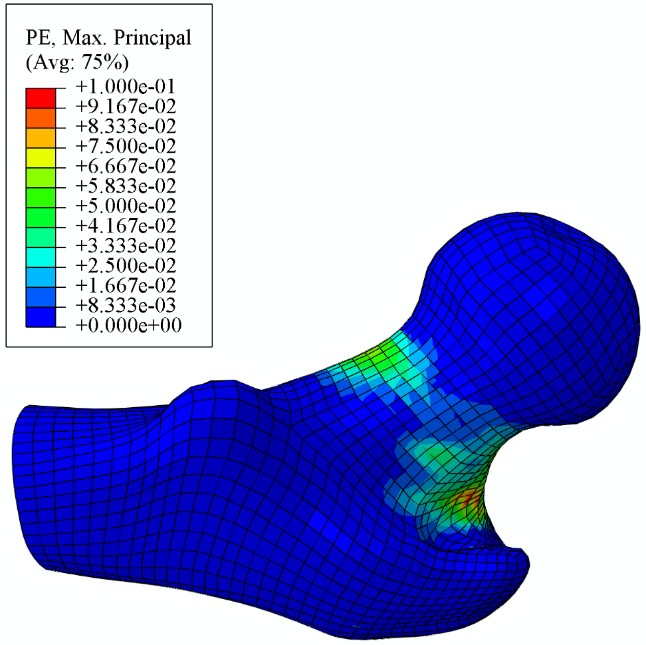 | 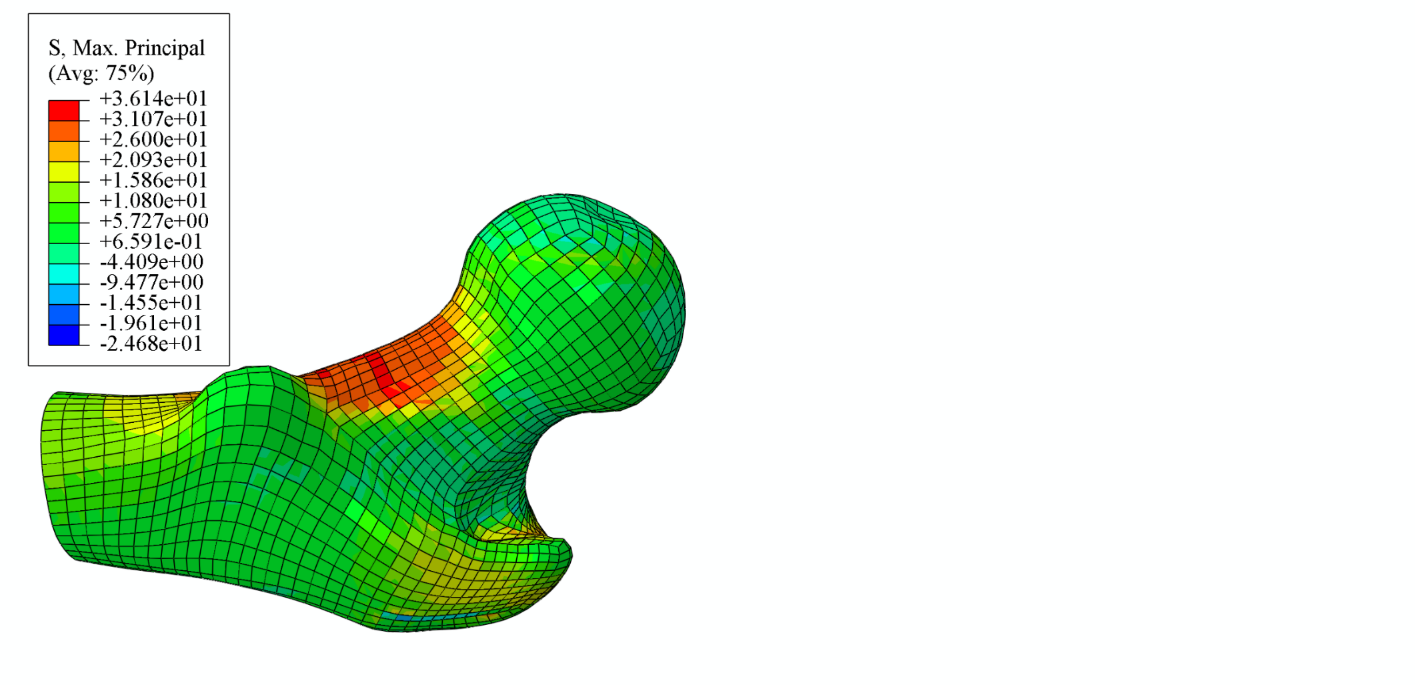 |
| --- | --- |
| (a) | (b) |

Fig. C1. 3D-DXA-based FE results for one randomly selected femur under side fall configuration at maximum reaction force: maximum principal plastic strain distribution (a) and maximum principal stress distribution (b)

**Appendix D**

The mechanical descriptors obtained from FE analysis for the complete dataset of control and fracture subjects are shown in Tables D1 and D2, respectively.

Table D1. FE-derived mechanical descriptors in control subjects

| Subjects | Strength (N) | Displacement (mm) | Nonlinear Energy (N.mm) | Linear Elastic Force (N) | Linear Elastic Displacement (mm) | Linear elastic energy (N.mm) | Nonlinear Deformation (mm) | Residual Displacement (mm) | Dissipated Energy (N.mm) | Residual Energy (N.mm) |
| --- | --- | --- | --- | --- | --- | --- | --- | --- | --- | --- |
| 1 | 3580.70 | 2.53 | 6394.58 | 2751.30 | 0.93 | 1274.23 | 1.60 | 1.32 | 4200.03 | 2158.28 |
| 2 | 3515.83 | 3.20 | 8346.88 | 2598.46 | 0.94 | 1224.40 | 2.26 | 1.92 | 5891.68 | 2241.54 |
| 3 | 3302.19 | 2.67 | 6365.87 | 2564.71 | 0.94 | 1199.61 | 1.73 | 1.47 | 4305.03 | 1988.69 |
| 4 | 3072.47 | 2.67 | 6009.66 | 2437.39 | 0.90 | 1098.48 | 1.77 | 1.53 | 4231.47 | 1745.49 |
| 5 | 4084.88 | 3.07 | 9104.55 | 3196.15 | 1.06 | 1691.05 | 2.01 | 1.72 | 6259.54 | 2762.25 |
| 6 | 3012.21 | 2.93 | 6510.44 | 2465.31 | 0.99 | 1221.54 | 1.94 | 1.72 | 4714.49 | 1823.61 |
| 7 | 2804.13 | 3.47 | 7148.76 | 2014.38 | 0.99 | 997.76 | 2.48 | 2.09 | 5043.77 | 1933.47 |
| 8 | 3748.49 | 2.40 | 6508.01 | 3070.33 | 0.88 | 1354.42 | 1.52 | 1.32 | 4518.22 | 2018.82 |
| 9 | 3474.95 | 2.67 | 6808.87 | 2755.07 | 0.90 | 1234.67 | 1.77 | 1.54 | 4802.61 | 1964.18 |
| 10 | 3961.94 | 3.20 | 9385.28 | 2908.59 | 0.94 | 1363.96 | 2.26 | 1.92 | 6612.83 | 2530.78 |
| 11 | 3528.04 | 2.53 | 6533.42 | 2871.13 | 0.89 | 1270.81 | 1.64 | 1.44 | 4622.53 | 1918.86 |
| 12 | 4170.97 | 2.93 | 8994.05 | 3425.61 | 1.01 | 1737.39 | 1.92 | 1.69 | 6445.57 | 2575.71 |
| 13 | 2672.48 | 2.67 | 5194.62 | 2088.70 | 0.90 | 935.58 | 1.77 | 1.52 | 3633.49 | 1531.65 |
| 14 | 3541.97 | 2.53 | 6448.20 | 2808.48 | 0.90 | 1270.61 | 1.63 | 1.39 | 4417.16 | 2020.98 |
| 15 | 2426.91 | 2.40 | 4151.45 | 1943.73 | 0.90 | 872.47 | 1.50 | 1.28 | 2800.08 | 1360.15 |
| 16 | 3574.38 | 3.07 | 8067.49 | 2824.85 | 1.00 | 1414.03 | 2.07 | 1.80 | 5776.76 | 2263.97 |
| 17 | 4015.58 | 2.13 | 6174.67 | 3222.14 | 0.78 | 1264.59 | 1.35 | 1.15 | 4178.42 | 1964.06 |
| 18 | 4340.43 | 2.53 | 7912.06 | 3475.87 | 0.93 | 1618.70 | 1.60 | 1.37 | 5350.58 | 2524.09 |
| 19 | 2764.21 | 2.67 | 5319.93 | 2138.65 | 0.93 | 991.50 | 1.74 | 1.47 | 3612.71 | 1656.35 |
| 20 | 3700.62 | 2.40 | 6382.15 | 3006.12 | 0.90 | 1351.57 | 1.50 | 1.29 | 4344.02 | 2048.22 |
| 21 | 4447.62 | 2.53 | 8103.30 | 3540.36 | 0.91 | 1616.54 | 1.62 | 1.38 | 5531.62 | 2551.22 |
| 22 | 4142.41 | 2.40 | 7210.01 | 3412.68 | 0.89 | 1512.07 | 1.51 | 1.32 | 5011.92 | 2227.86 |
| 23 | 3415.75 | 2.53 | 6371.57 | 2834.84 | 0.90 | 1277.41 | 1.63 | 1.44 | 4521.06 | 1854.58 |
| 24 | 3002.83 | 2.80 | 6073.30 | 2392.55 | 1.00 | 1190.89 | 1.80 | 1.55 | 4188.48 | 1875.91 |
| 25 | 3780.20 | 2.67 | 7279.66 | 2931.54 | 0.93 | 1360.89 | 1.74 | 1.47 | 4949.55 | 2262.88 |
| 26 | 3244.07 | 2.53 | 5849.83 | 2517.09 | 0.90 | 1135.33 | 1.63 | 1.37 | 3945.79 | 1885.84 |
| 27 | 3948.20 | 2.67 | 7593.44 | 3063.37 | 0.94 | 1446.83 | 1.73 | 1.45 | 5099.53 | 2403.34 |
| 28 | 3493.78 | 2.93 | 7496.05 | 2805.76 | 0.98 | 1378.89 | 1.95 | 1.71 | 5381.77 | 2138.06 |
| 29 | 2658.34 | 2.80 | 5448.65 | 2138.03 | 0.95 | 1013.36 | 1.85 | 1.62 | 3894.90 | 1566.59 |
| 30 | 2480.43 | 2.67 | 4817.43 | 1934.47 | 0.90 | 867.13 | 1.77 | 1.52 | 3361.95 | 1425.65 |
| 31 | 3008.13 | 2.80 | 6100.31 | 2407.31 | 0.99 | 1187.78 | 1.81 | 1.57 | 4248.10 | 1854.67 |
| 32 | 3417.49 | 2.93 | 7366.56 | 2776.10 | 0.98 | 1363.80 | 1.95 | 1.72 | 5335.30 | 2066.78 |
| 33 | 3345.62 | 2.40 | 5775.68 | 2717.17 | 0.89 | 1207.23 | 1.51 | 1.31 | 3965.72 | 1830.25 |
| 34 | 4485.79 | 3.07 | 10184.04 | 3603.05 | 1.01 | 1826.54 | 2.06 | 1.81 | 7319.84 | 2831.18 |
| 35 | 3567.74 | 2.13 | 5396.95 | 2783.72 | 0.79 | 1102.26 | 1.34 | 1.12 | 3550.00 | 1810.58 |
| 36 | 2711.45 | 2.67 | 5294.97 | 2143.12 | 0.90 | 966.76 | 1.77 | 1.53 | 3716.14 | 1547.50 |
| 37 | 3122.13 | 3.33 | 7594.77 | 2465.04 | 1.11 | 1371.98 | 2.22 | 1.92 | 5369.39 | 2200.90 |
| 38 | 3407.84 | 2.53 | 6264.20 | 2748.72 | 0.91 | 1245.90 | 1.62 | 1.41 | 4335.36 | 1915.05 |
| 39 | 3467.16 | 2.40 | 5982.76 | 2821.58 | 0.90 | 1262.94 | 1.50 | 1.30 | 4094.79 | 1906.97 |
| 40 | 4511.03 | 2.53 | 8174.19 | 3534.92 | 0.91 | 1606.31 | 1.62 | 1.37 | 5521.57 | 2615.91 |
| 41 | 3295.31 | 2.67 | 6369.70 | 2579.56 | 0.94 | 1206.22 | 1.73 | 1.48 | 4339.62 | 1968.46 |
| 42 | 2386.28 | 2.67 | 4570.74 | 1823.45 | 0.93 | 850.46 | 1.74 | 1.45 | 3054.79 | 1456.50 |
| 43 | 3160.91 | 3.33 | 7772.84 | 2502.77 | 1.06 | 1328.80 | 2.27 | 1.99 | 5638.79 | 2119.54 |
| 44 | 3216.84 | 2.40 | 5413.90 | 2531.25 | 0.94 | 1187.90 | 1.46 | 1.21 | 3475.35 | 1918.53 |
| 45 | 3120.32 | 2.93 | 6657.79 | 2495.41 | 1.00 | 1243.14 | 1.93 | 1.68 | 4734.68 | 1943.72 |
| 46 | 3408.29 | 2.53 | 6343.87 | 2804.11 | 0.89 | 1241.12 | 1.64 | 1.45 | 4524.34 | 1833.55 |
| 47 | 3176.21 | 2.53 | 5863.89 | 2575.10 | 0.89 | 1147.87 | 1.64 | 1.43 | 4120.12 | 1746.32 |
| 48 | 3825.05 | 2.40 | 6628.43 | 3130.16 | 0.89 | 1394.79 | 1.51 | 1.31 | 4567.65 | 2082.82 |
| 49 | 4350.86 | 2.27 | 7076.42 | 3433.99 | 0.83 | 1427.79 | 1.44 | 1.22 | 4744.57 | 2292.01 |
| 50 | 2896.74 | 2.53 | 5392.15 | 2369.91 | 0.87 | 1031.79 | 1.66 | 1.47 | 3866.80 | 1541.52 |
| 51 | 3701.84 | 2.40 | 6319.41 | 2998.89 | 0.94 | 1409.33 | 1.46 | 1.24 | 4160.36 | 2147.46 |
| 52 | 3183.35 | 2.67 | 6141.72 | 2479.25 | 0.93 | 1148.21 | 1.74 | 1.48 | 4198.54 | 1892.99 |
| 53 | 3723.19 | 2.53 | 6873.42 | 2997.12 | 0.87 | 1310.83 | 1.66 | 1.44 | 4858.61 | 2022.87 |
| 54 | 3111.54 | 2.67 | 6191.67 | 2518.24 | 0.86 | 1080.27 | 1.81 | 1.61 | 4540.93 | 1649.26 |
| 55 | 3400.57 | 2.53 | 6316.31 | 2753.24 | 0.86 | 1177.50 | 1.67 | 1.47 | 4543.28 | 1796.27 |
| 56 | 3221.55 | 2.53 | 5831.02 | 2529.78 | 0.92 | 1158.55 | 1.61 | 1.36 | 3927.44 | 1878.79 |
| 57 | 4926.34 | 2.27 | 8169.75 | 4006.14 | 0.81 | 1630.01 | 1.46 | 1.27 | 5680.32 | 2464.83 |
| 58 | 3220.68 | 2.67 | 6147.95 | 2465.12 | 0.95 | 1172.68 | 1.72 | 1.43 | 4062.47 | 2001.69 |
| 59 | 3171.76 | 2.53 | 5901.64 | 2585.27 | 0.86 | 1114.54 | 1.67 | 1.47 | 4245.64 | 1677.58 |
| 60 | 3760.60 | 2.67 | 7392.90 | 3013.73 | 0.90 | 1362.68 | 1.77 | 1.54 | 5228.99 | 2121.78 |
| 61 | 2598.24 | 2.67 | 5122.12 | 2100.69 | 0.91 | 952.49 | 1.76 | 1.55 | 3643.13 | 1457.12 |
| 62 | 2214.68 | 2.80 | 4499.54 | 1762.82 | 0.96 | 847.99 | 1.84 | 1.59 | 3169.50 | 1338.44 |
| 63 | 2697.16 | 3.07 | 6002.82 | 2073.14 | 1.01 | 1045.58 | 2.06 | 1.76 | 4198.12 | 1769.75 |
| 64 | 3373.14 | 2.67 | 6523.94 | 2622.79 | 0.92 | 1205.64 | 1.75 | 1.49 | 4466.84 | 1994.14 |

Table D2. FE-derived mechanical descriptors in fracture subjects

| Subjects | Strength (N) | Displacement (mm) | Nonlinear Energy (N.mm) | Linear Elastic Force (N) | Linear Elastic Displacement (mm) | Linear elastic energy (N.mm) | Nonlinear Deformation (mm) | Residual Displacement (mm) | Dissipated Energy (N.mm) | Residual Energy (N.mm) |
| --- | --- | --- | --- | --- | --- | --- | --- | --- | --- | --- |
| 1 | 2869.09 | 2.93 | 6178.84 | 2364.33 | 1.03 | 1219.39 | 1.90 | 1.68 | 4396.84 | 1795.62 |
| 2 | 2726.32 | 2.27 | 4452.22 | 2157.05 | 0.82 | 883.53 | 1.45 | 1.23 | 3020.86 | 1411.41 |
| 3 | 2798.51 | 2.93 | 5958.55 | 2249.71 | 1.03 | 1157.15 | 1.90 | 1.65 | 4170.96 | 1790.57 |
| 4 | 3895.05 | 3.20 | 9195.67 | 2843.41 | 0.94 | 1333.43 | 2.26 | 1.92 | 6460.80 | 2502.18 |
| 5 | 3086.49 | 2.93 | 6659.35 | 2531.72 | 1.01 | 1273.75 | 1.92 | 1.70 | 4790.99 | 1893.14 |
| 6 | 3408.23 | 2.93 | 7483.22 | 2806.28 | 0.92 | 1287.58 | 2.01 | 1.82 | 5651.47 | 1899.19 |
| 7 | 2981.22 | 3.20 | 7094.13 | 2219.77 | 0.94 | 1039.89 | 2.26 | 1.94 | 5055.45 | 1875.68 |
| 8 | 3289.70 | 2.80 | 6700.94 | 2639.89 | 0.97 | 1284.49 | 1.83 | 1.59 | 4711.90 | 1994.67 |
| 9 | 2584.71 | 3.60 | 7054.45 | 2118.84 | 1.06 | 1118.01 | 2.54 | 2.31 | 5445.84 | 1663.70 |
| 10 | 3332.71 | 2.80 | 6787.29 | 2662.18 | 0.97 | 1286.75 | 1.83 | 1.59 | 4772.77 | 2016.57 |
| 11 | 1813.48 | 3.20 | 4234.06 | 1292.68 | 0.96 | 618.44 | 2.24 | 1.86 | 2888.79 | 1217.15 |
| 12 | 2223.84 | 3.07 | 5070.72 | 1777.37 | 0.98 | 867.98 | 2.09 | 1.85 | 3702.45 | 1358.83 |
| 13 | 3109.41 | 2.67 | 6030.96 | 2418.71 | 0.90 | 1085.27 | 1.77 | 1.52 | 4198.68 | 1793.61 |
| 14 | 2924.92 | 3.47 | 7521.46 | 2172.60 | 1.05 | 1137.09 | 2.42 | 2.06 | 5257.50 | 2060.93 |
| 15 | 3717.14 | 3.07 | 8350.17 | 2948.84 | 1.05 | 1542.79 | 2.02 | 1.75 | 5842.61 | 2451.45 |
| 16 | 3046.39 | 2.93 | 6710.08 | 2571.13 | 0.96 | 1235.69 | 1.97 | 1.79 | 5038.17 | 1734.73 |
| 17 | 4137.94 | 3.20 | 10018.99 | 2940.99 | 0.82 | 1198.81 | 2.38 | 2.05 | 7278.08 | 2373.18 |
| 18 | 1790.76 | 3.87 | 5273.87 | 1464.94 | 1.10 | 805.50 | 2.77 | 2.53 | 4116.33 | 1203.65 |
| 19 | 3277.77 | 2.53 | 6097.89 | 2674.14 | 0.87 | 1160.87 | 1.66 | 1.47 | 4370.79 | 1744.10 |
| 20 | 2901.91 | 2.93 | 6155.50 | 2314.26 | 1.03 | 1190.43 | 1.90 | 1.64 | 4282.62 | 1871.74 |
| 21 | 2569.53 | 2.93 | 5547.41 | 2077.35 | 0.96 | 993.66 | 1.97 | 1.75 | 4065.41 | 1520.29 |
| 22 | 2826.47 | 2.80 | 5734.11 | 2254.08 | 0.98 | 1105.49 | 1.82 | 1.57 | 3993.87 | 1738.23 |
| 23 | 2528.53 | 3.47 | 6457.24 | 1824.57 | 1.01 | 919.52 | 2.46 | 2.07 | 4517.09 | 1765.94 |
| 24 | 3631.80 | 2.80 | 7516.75 | 2971.06 | 0.94 | 1393.35 | 1.86 | 1.65 | 5467.37 | 2082.00 |
| 25 | 3250.85 | 2.80 | 6747.35 | 2674.89 | 0.94 | 1256.04 | 1.86 | 1.66 | 4921.40 | 1855.19 |
| 26 | 2950.89 | 2.80 | 6098.35 | 2407.42 | 0.94 | 1127.08 | 1.86 | 1.65 | 4434.21 | 1693.39 |
| 27 | 2817.81 | 2.93 | 6027.35 | 2271.59 | 1.01 | 1141.71 | 1.92 | 1.68 | 4288.67 | 1756.79 |
| 28 | 2899.04 | 2.93 | 6163.96 | 2298.02 | 1.01 | 1155.01 | 1.92 | 1.66 | 4324.41 | 1838.18 |
| 29 | 2935.25 | 3.07 | 6625.88 | 2341.55 | 1.03 | 1204.89 | 2.04 | 1.78 | 4701.48 | 1893.34 |
| 30 | 3684.54 | 2.93 | 7896.61 | 2988.87 | 1.02 | 1520.47 | 1.91 | 1.68 | 5598.37 | 2310.62 |
| 31 | 3092.18 | 3.07 | 6993.07 | 2505.18 | 1.06 | 1333.57 | 2.01 | 1.76 | 4919.52 | 2031.74 |
| 32 | 2490.67 | 2.80 | 5039.18 | 1975.87 | 0.97 | 961.10 | 1.83 | 1.57 | 3519.68 | 1527.16 |
| 33 | 3046.94 | 2.93 | 6550.75 | 2466.04 | 0.99 | 1217.05 | 1.94 | 1.71 | 4721.19 | 1857.95 |
| 34 | 2820.68 | 2.80 | 5793.18 | 2295.18 | 0.96 | 1104.50 | 1.84 | 1.62 | 4142.42 | 1668.17 |
| 35 | 2424.01 | 2.53 | 4526.35 | 1998.39 | 0.87 | 869.03 | 1.66 | 1.48 | 3268.00 | 1278.63 |
| 36 | 5060.06 | 2.53 | 9276.28 | 4105.52 | 0.93 | 1914.57 | 1.60 | 1.38 | 6335.02 | 2908.35 |
| 37 | 2092.69 | 3.60 | 5567.15 | 1660.42 | 1.12 | 931.56 | 2.48 | 2.19 | 4106.23 | 1479.72 |
| 38 | 2674.20 | 2.80 | 5545.24 | 2207.89 | 0.96 | 1057.65 | 1.84 | 1.64 | 4008.43 | 1551.59 |
| 39 | 3537.32 | 3.47 | 9032.45 | 2574.97 | 1.05 | 1346.99 | 2.42 | 2.03 | 6217.92 | 2541.97 |
| 40 | 2821.52 | 3.51 | 7032.42 | 1981.63 | 1.09 | 1082.93 | 2.42 | 1.95 | 4696.43 | 2195.45 |
| 41 | 2892.26 | 3.47 | 7495.60 | 1996.10 | 0.91 | 912.46 | 2.56 | 2.15 | 5249.48 | 1915.68 |
| 42 | 2841.10 | 2.93 | 6042.27 | 2249.98 | 1.01 | 1131.92 | 1.92 | 1.66 | 4230.21 | 1804.82 |
| 43 | 3151.47 | 2.80 | 6347.12 | 2474.69 | 0.98 | 1212.90 | 1.82 | 1.55 | 4370.92 | 1967.02 |
| 44 | 2568.91 | 2.67 | 4961.16 | 1984.28 | 0.91 | 901.47 | 1.76 | 1.49 | 3405.50 | 1510.92 |
| 45 | 2984.67 | 3.33 | 7335.06 | 2354.95 | 1.06 | 1244.24 | 2.27 | 1.99 | 5321.09 | 1998.64 |
| 46 | 3452.44 | 2.67 | 6748.39 | 2734.12 | 0.90 | 1233.81 | 1.77 | 1.53 | 4742.18 | 1967.26 |
| 47 | 3459.65 | 2.80 | 7108.19 | 2819.64 | 0.97 | 1363.39 | 1.83 | 1.61 | 5072.25 | 2052.57 |
| 48 | 2332.90 | 2.93 | 4994.29 | 1845.20 | 0.96 | 887.18 | 1.97 | 1.71 | 3587.37 | 1418.12 |
| 49 | 2425.62 | 3.20 | 5690.72 | 1744.81 | 0.94 | 821.64 | 2.26 | 1.89 | 3947.14 | 1587.93 |
| 50 | 2889.73 | 2.67 | 5691.41 | 2319.50 | 0.89 | 1036.12 | 1.78 | 1.56 | 4061.56 | 1608.18 |
| 51 | 3047.52 | 2.93 | 6550.76 | 2474.58 | 1.00 | 1235.86 | 1.93 | 1.70 | 4699.64 | 1874.39 |
| 52 | 3700.51 | 2.93 | 7916.03 | 2992.83 | 1.02 | 1527.02 | 1.91 | 1.67 | 5589.79 | 2334.55 |
| 53 | 2681.58 | 3.20 | 6371.11 | 1988.64 | 0.94 | 934.48 | 2.26 | 1.93 | 4518.10 | 1699.18 |
| 54 | 2970.42 | 2.93 | 6375.42 | 2390.02 | 0.98 | 1175.09 | 1.95 | 1.71 | 4584.08 | 1815.11 |
| 55 | 3727.01 | 3.47 | 9748.61 | 2655.44 | 0.92 | 1225.72 | 2.55 | 2.17 | 6945.83 | 2414.57 |
| 56 | 2443.82 | 2.67 | 4760.51 | 1926.74 | 0.91 | 880.26 | 1.76 | 1.51 | 3306.82 | 1416.13 |
| 57 | 2949.83 | 2.53 | 5447.94 | 2385.20 | 0.88 | 1052.22 | 1.65 | 1.44 | 3845.61 | 1609.35 |
| 58 | 3679.08 | 2.67 | 7092.79 | 2855.56 | 0.93 | 1332.12 | 1.74 | 1.47 | 4802.85 | 2211.27 |
| 59 | 3506.42 | 2.53 | 6535.76 | 2886.24 | 0.88 | 1270.45 | 1.65 | 1.46 | 4675.20 | 1875.07 |
| 60 | 2315.48 | 3.07 | 5239.24 | 1844.54 | 1.00 | 923.69 | 2.07 | 1.81 | 3775.68 | 1455.58 |
| 61 | 2359.25 | 2.93 | 5067.54 | 1934.28 | 1.02 | 991.24 | 1.91 | 1.68 | 3610.73 | 1474.65 |
| 62 | 3224.14 | 3.07 | 7166.64 | 2494.52 | 1.04 | 1293.18 | 2.03 | 1.73 | 4952.08 | 2160.30 |
| 63 | 2434.73 | 3.07 | 5585.71 | 1972.54 | 0.97 | 958.46 | 2.10 | 1.87 | 4127.77 | 1460.23 |
| 64 | 2919.35 | 3.20 | 6831.67 | 2101.27 | 0.96 | 1012.85 | 2.24 | 1.86 | 4675.95 | 1955.04 |

**Appendix E**

The effect of different fall directions on the discrimination ability of the FE-derived strength and best-performing descriptors involving the inelastic model response is presented in Table E1.

Table E1. The effect of different fall directions on the discrimination performance of FE-derived strength and best performing descriptors involving the inelastic model response

| Internal/Adduction Rotation | AUROC (p-value vs. aBMD) | | | |
| --- | --- | --- | --- | --- |
|  | Strength | Strength + Residual Displacement | Linear Elastic Force + Residual Displacement | Residual Displacement |
| $0^{^{\circ}}/0^{^{\circ}}$ | 0.72 (0.305) | 0.77 (0.012) | 0.80$(<0.001$ ) | 0.75 (0.162) |
| $0^{^{\circ}}/{15}^{^{\circ}}$ | 0.71 (0.526) | 0.77 (0.009) | 0.76 (0.030 ) | 0.75 (0.189) |
| $0^{^{\circ}}/{30}^{^{\circ}}$ | 0.69 (0.935) | 0.75 (0.125) | 0.74 (0.283) | 0.74 (0.419) |
| $5^{^{\circ}}/5^{^{\circ}}$ | 0.72 (0.275) | 0.80 (0.001) | 0.79 (0.005) | 0.79 (0.033) |
| ${15}^{^{\circ}}/0^{^{\circ}}$ | 0.70 (0.818) | 0.74 (0.087) | 0.74 (0.093) | 0.73 (0.374) |
| ${15}^{^{\circ}}/{10}^{^{\circ}}$ | 0.70 (0.870) | 0.76 (0.036) | 0.77 (0.010) | 0.75 (0.228) |
| ${15}^{^{\circ}}/{15}^{^{\circ}}$ | 0.70 (0.894) | 0.74 (0.083) | 0.74 (0.057) | 0.73 (0.394) |
| ${15}^{^{\circ}}/{30}^{^{\circ}}$ | 0.69 (0.843) | 0.71 (0.503) | 0.71 (0.430) | 0.70 (0.916) |
| ${30}^{^{\circ}}/0^{^{\circ}}$ | 0.68 (0.604) | 0.70 (0.827) | 0.69 (0.918) | 0.70 (0.931) |
| ${30}^{^{\circ}}/{15}^{^{\circ}}$ | 0.68 (0.697) | 0.71 (0.537) | 0.71 (0.503) | 0.70 (0.877) |
| ${30}^{^{\circ}}/{30}^{^{\circ}}$ | 0.68 (0.679) | 0.71 (0.560) | 0.71 (0.520) | 0.70 (0.863) |
